# Performance characteristics of an antibody-based multiplex kit for determining recent HIV-1 infection

**DOI:** 10.1371/journal.pone.0176593

**Published:** 2017-05-04

**Authors:** Kelly A. Curtis, Debra L. Hanson, Krystin Ambrose Price, S. Michele Owen

**Affiliations:** Division of HIV/AIDS Prevention, National Center for HIV/AIDS, Hepatitis, STD, and TB Prevention, Centers for Disease Control and Prevention, Atlanta, GA, United States of America; California State University Fresno, UNITED STATES

## Abstract

The availability of reliable laboratory methods for determining recent HIV infection is vital for accurate estimation of population-based incidence. The mean duration of recent infection (MDRI) and false recent rate (FRR) are critical parameters for HIV incidence assays, as they impact HIV incidence estimates and provide a measure of assay performance. The HIV-1 Multiplex assay is an in-house developed, magnetic bead-based assay that measures virus-specific antibody levels and avidity to multiple analytes. To ensure quality control and to facilitate transfer of the assay to external laboratories or testing facilities, the in-house assay has been adapted and produced in kit form. Here, we describe the performance characteristics of the multiplex kit and demonstrate the stability of the kit components over a one-year period. Two statistical methods were employed to estimate the MDRI of the individual analytes and five different algorithms, combining multiple analyte values. The MDRI estimates for the individual analytes and five algorithms were all between 200 and 300 days post-seroconversion, with no notable difference between the two statistical approaches. All five algorithms exhibited a 0% FRR with specimens from long-term, subtype B HIV-1-infected individuals. The assay parameters described in this study provide the necessary tools to implement the HIV-1 multiplex assay and improves the utility of the assay for field use.

## Introduction

Estimation of HIV incidence, the rate of new infections in a population, is a vital public health tool for evaluating the efficacy of intervention measures, monitoring recent transmission, and identifying high-risk populations. Laboratory methods for determining recent HIV infection have become an integral component in obtaining incidence estimates, since they are less costly than follow-up studies, can be applied to cross-sectional collections of specimens, and the assays are relatively easy to perform. Since the pioneer study describing the use of a laboratory assay for determining recent infection [1], several reviews have been published to highlight the technology and underscore the importance and unique challenges associated with incidence assays [2–5]. Generally, HIV incidence assays fall into two categories: [1] developed specifically for the purpose of estimating incidence, such as the BED-CEIA and HIV-1 Limiting Antigen (LAg)-Avidity EIA (Sedia Biosciences Corp., Portland, OR)[6, 7] or [2] adapted or modified from commercial diagnostic tests, such as the Bio-Rad Avidity assay [8]. The majority of HIV incidence assays measure primarily HIV-specific antibody titer or avidity, which increase gradually over time from infection and allow for differentiation between recent and long-term infection [7, 9–14]. Although several non-serological methods are in the early stages of development [15–17], the benefits of serological biomarkers continue to support the use of antibody-based assays for determining recent infection. Antibody is a remarkably stable biomarker in plasma or dried blood spots (DBS) and levels of virus-specific antibody follow a relatively predictable pattern of maturation post-seroconversion [13, 18]. However, there are well-documented limitations associated with antibody-based incidence assays; e.g. factors that lead to virus suppression (natural or antiretroviral-induced) and certain subtypes are associated with increased misclassification or high false-recent rates (FRR) [3, 5].

HIV-1 incidence in the United States has been estimated by testing cross-sectional sero-surveillance specimens with the BED-CEIA and LAg assays [19–21]. Such assays have been instrumental in monitoring changes in the number of new HIV infections over time, however, the accuracy of incidence assays have been questioned due to reports of overestimation of incidence [2, 22, 23]. One approach to overcoming the limitations associated with these assays is employing the use of a recent infection testing algorithm (RITA) or multi-assay algorithm (MAA), involving one or more incidence assays along with clinical data (CD4+ T-cell count, viral load, etc.)[24]. The algorithm-based method has shown improved performance and reduced FRRs as compared to a stand-alone incidence assay [24–26]. Similarly, improved FRRs and incidence estimates have been demonstrated using multi-analyte algorithms based on three or more measures within a single assay platform, the HIV-1 Multiplex assay [27]. The Multiplex assay is a magnetic bead-based assay, analyzed on the Bio-Plex system (Bio-Rad Laboratories, Hercules, CA), which measures virus-specific antibody levels and antibody avidity to multiple analytes [13, 27]. Antibody avidity is measured through treatment of the antibody-bead complex with the dissociative agent, diethylamine (DEA). Previous studies have demonstrated that both HIV antibody levels and avidity, particularly to envelope antigens, increase steadily post-seroconversion and provide a clear, measureable distinction in assay reactivity between recent and long-term infection [13, 27]. Furthermore, multi-algorithms, based on combinations of 3 to 6 analytes within the HIV-1 Multiplex assay, improves assay performance relative to each individual analyte [27]. Recently, the in-house developed assay was adapted to a kit to facilitate transfer of the incidence assay to other laboratories or testing sites [28].

According to the target product profile (TPP) for HIV incidence assays, the desired FRR for any incidence assay is less than 2% [29]. Given that FRR must be specific for the population under evaluation and accurate estimates are difficult to obtain, the ideal HIV incidence assay would have a FRR close to or equal to 0%. Additionally, the mean duration of recent infection (MDRI), the average time between seroconversion and reaching a specified biomarker cutoff value, is a critical assay measure, which has a significant impact on incidence assay performance. Statistical methods for estimating MDRI have evolved and, therefore, recalibrations of these measures are necessary [8, 30]. A multiplexed or algorithm-based approach of determining recent infection offers additional challenges, since FRR and MDRI estimates are unique to each algorithm, the scales of various analytes differ, and the analytes may be highly correlated. Here, we describe the performance characteristics of the HIV-1 Multiplex kit, using two statistical methods for estimating MDRI and combining multiple analyte values. The FRR of each analyte or combination of analytes is also reported. Furthermore, the stability of the kit components was evaluated over a one-year period.

## Materials and methods

### Specimens

For defining cutoff values and subsequent estimation of MDRIs for the HIV-1 Multiplex Assay based upon selected cutoffs, 608 specimens from 95 ART-naïve, subtype B HIV-1-infected subjects were evaluated. Longitudinal specimens from recent HIV-1 seroconverters were obtained commercially or from previous studies, described in detail elsewhere [13, 27, 31]. Briefly, five HIV-1 seroconversion panels (n = 44) were purchased from Zeptometrix Corp. (Buffalo, NY) and three panels (n = 15) were obtained from SeraCare Life Sciences, Inc. (Milford, MA). Longitudinal specimens from 48 recent seroconverters (n = 302) were collected as part of the Vaccine Preparedness Study for the HIV Network for Prevention Trials (HIVNET; ClinicalTrials.gov identifier NCT00000915) [32, 33]. Specimens from 30 subjects (n = 166) were obtained from the AIDSVAX B/B Phase III Vaccine Trial (VAX004; ClinicalTrials.gov identifier NCT00002441) [34]. A total of nine longitudinal seroconversion panels (n = 81) were obtained through the Seroconversion Incidence Panel Project (SIPP) in collaboration with SeraCare Life Sciences, Inc. [18]. Inclusion criteria for all seroconversion panels selected for this study were as follows: [1] each panel must have at least 4 consecutive observations, and [2] the interval of time between the last negative and first positive antibody test must be <240 days. The total follow up time for the majority of the study subjects included in this study was ≥ one year.

For estimation of FRR, 296 specimens from long-term HIV-1-infected subjects from a historic prospective study were evaluated. The archived plasma specimens were obtained from a study involving subtype B HIV-1-infected men who have sex with men (MSM), enrolled between 1982 and 1983 in Atlanta, Georgia [35–38]. The specimens included in this study were collected >2 years from the study entry date, since diagnostic test dates were not available for this cohort. Additionally, 38 specimens from the seroconversion panel data, collected >731 days after the mid-date between last negative and first positive tests, were included in the evaluation. All samples were unlinked from personal identifiers and determined not to be human subjects research by the Centers for Disease Control and Prevention.

### HIV-1 Multiplex assay

The in-house, CDC-developed HIV-1 Multiplex assay was adapted for kit production by Radix BioSolutions, Ltd. (Georgetown, TX), as described previously [28]. Specific kit components included: 10X magnetic bead mix, sample dilution buffer [13], assay buffer [13], 0.1M diethylamine (DEA) in assay buffer, and detection antibody (PE-conjugated, goat anti-human IgG). The bead mix was composed of bead sets conjugated to recombinant, full-length subtype B HIV-1 proteins, gp120 (IIIB), gp160 (IIIB), and gp41 (MN), as well as anti-IgG and bovine serum albumin (BSA), which were included as sample loading and non-specific binding controls, respectively. The calibrator and high/low positive controls were not included in the kit, as they are prepared at the CDC. A normalized mean fluorescent intensity (MFI) value (MFI-n or -n) was derived by dividing the MFI of each specimen by the MFI of the calibrator. An avidity index (AI or -a) was calculated for each specimen using the following formula:

(normalizedMFIvalueofDEAtreatedwell÷normalizedMFIvalueofbuffertreatedwell)*100.

The analyte gp41-n is not considered for determining recent infection, as antibodies to gp41 develop rapidly, providing poor discrimination between recent and long-term infection [13]. All kits used in this study were obtained from the same production lot.

### Kit stability

**Fig 1:**
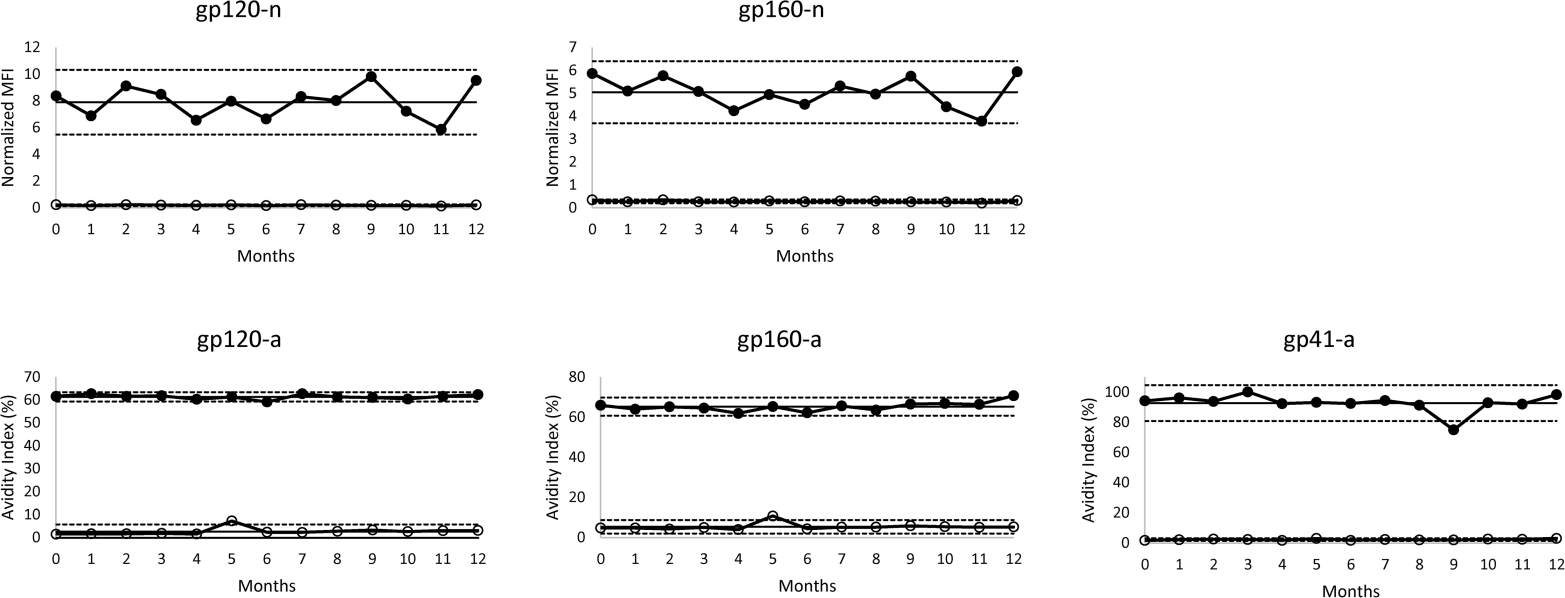
HIV-1 Multiplex assay kit stability. Levey-Jennings plots of high (closed circles) and low (open circles) positive controls over a 12 month period. The straight solid line represents the mean assay value of the high positive control, while the dashed lines represent 2 standard deviations above and below the mean.

Stability of the magnetic beads and kit assay reagents was assessed over a twelve month period. Upon receipt of the kits from the manufacturer, high and low positive controls were tested (t = 0). The controls were tested with kits from same production lot every month for up to 12 months. The high/low controls were obtained from a proficiency testing panel derived from HIV-1-positive serum [39]. The high positive control elicits assay values that are consistent with long-term infection, while the low control reacts as a recent infection. A Levey-Jennings chart was constructed to assess stability of the kit performance over the 12 month period (Fig 1).

### Determination of cutoff values

**Fig 2:**
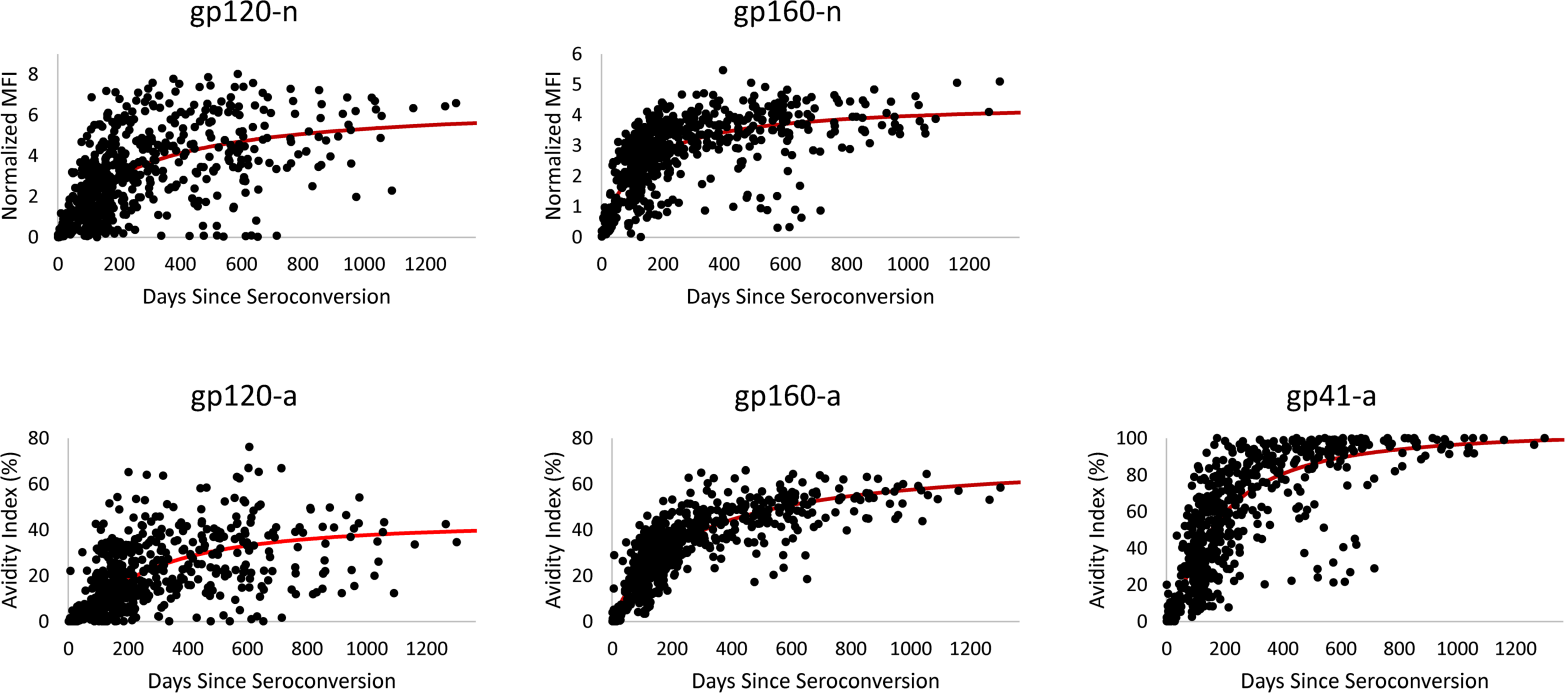
Longitudinal antibody responses to individual analytes. The MFI-n and AI values for 95 recent seroconverters (n = 608) were plotted over days since estimated seroconversion. The solid red lines represent prediction curves from fitting random intercept models implementing a 3-parameter function, avidity = $C/(1+{\left(\frac{days}{B}\right)}^{-A})$, for avidity measures and a 2-parameter function, OD = (*days* * *A*)/(*days* + *B*), for MFI-n values.

**Fig 3:**
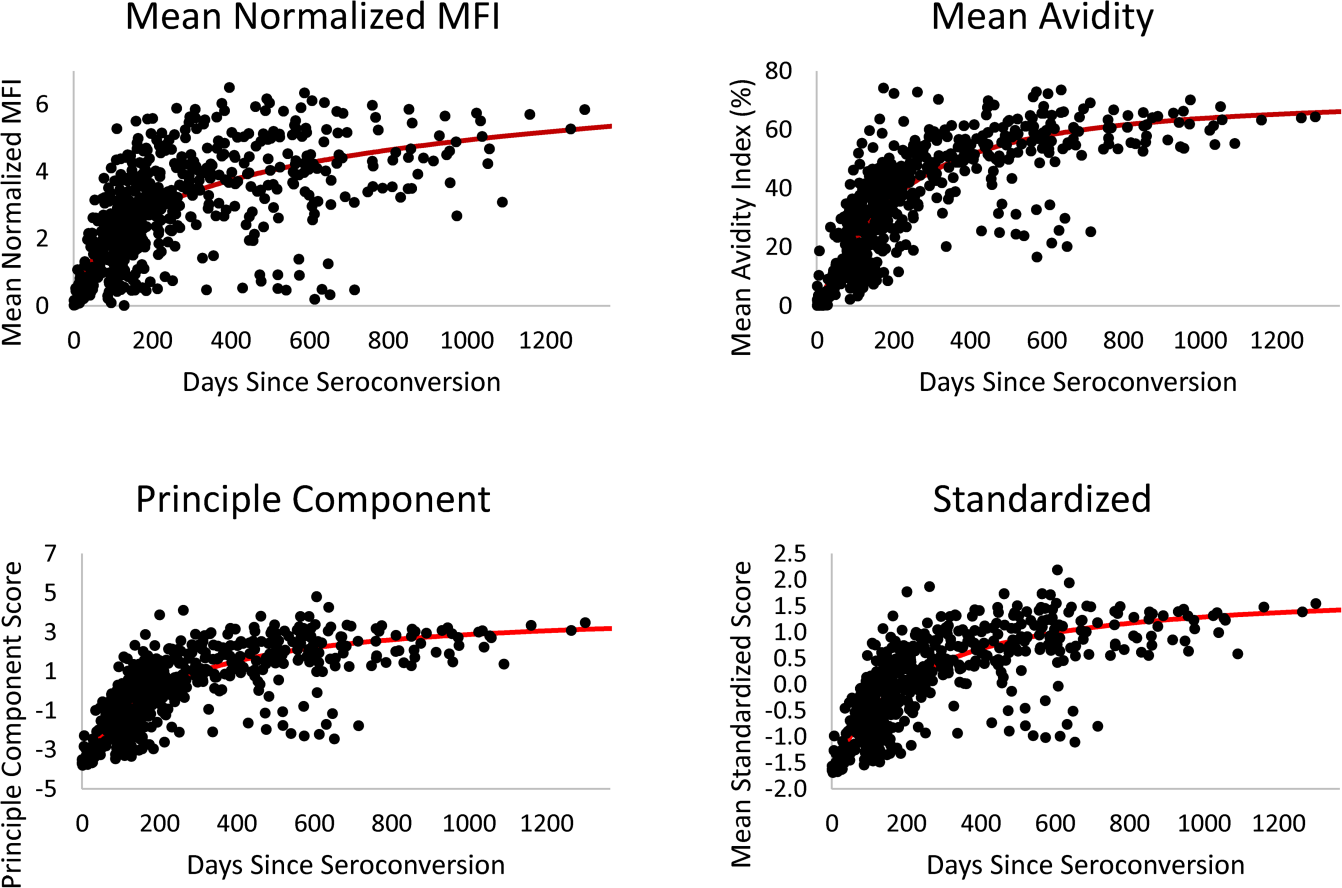
Intra-assay algorithms. The combined algorithm value for the mean normalized MFI, mean avidity index, principle component, and standardized score methods were plotted over days since estimated seroconversion. The solid red lines represent prediction curves from fitting random intercept models implementing a four-parameter function, score = $\left(\frac{A-D}{1+{\left(\frac{days}{C}\right)}^{B}}\right)+D$. Note, the multi-analyte algorithm is not included in the figure since the algorithm takes into account each individual analyte cutoff and does not generate a combined value or score.

The cutoff values used to distinguish recent from long-term infection for individual analytes and data reduction scores were defined as a level below the observed plateau following increasing values as graphically displayed in Figs 2 and 3. The tradeoff between longer recency duration with higher cutoff values and lower FRR with lower cutoff values motivated the selected criteria, e.g. with a lower cutoff value and shorter MDRI, the number of recent infections is less and the coefficient of variation in MDRI increases. However, with a higher cutoff value and longer MDRI, the FRR may be higher for individual analytes.

### Estimation of mean duration of recent infection (MDRI)

Methods for estimation of the MDRI were described in detail previously [27]. Briefly, two methods were used to calculate MDRI at specified recency cutoff values and within 2 years post-seroconversion of each analyte or combination of analytes. First, a Kaplan-Meier estimator of the survival function, describing the probability of being in the recent state as a function of time since detectable infection, was estimated after approximating entry time (detection) as the midpoint between last HIV-negative and first HIV-positive tests, and exit time (transition from recent to non-recent) using linear interpolation or regression on measurement readings that may fluctuate back and forth between the recent/non-recent states for each subject. If a non-recent result was not observed, the time in the recent state was right-censored at the subject’s last visit. Second, a binomial model of the probability of testing recent as a function of time since detection was modeled by fitting a binary value for the observed non-recent/recent classifications. Detection time was approximated as described for the survival method and was log-transformed prior to model fitting. To account for potential subject-level clustering effect on estimates of MDRI, a random intercept binomial model, with the logit parametric form was fit to the data. Bootstrapping was performed by sampling from subjects; 2000 replicates were computed.

### Combination algorithm and component scores

Highly correlated analytes assayed as part of the Bio-Plex system are challenging to consider individually in studies planned for estimation of recent infection. We therefore sought methods to better characterize the individual analyte values as either a multi-analyte algorithm based on a combination of analytes or as a reduced number of component scores, with both approaches resulting in a new value or score that may subsequently be used to test for recent HIV infection. Because performance of multi-analyte algorithms was assessed previously [27], we included only results for the ‘3/5 combination’ for comparison as a representative of the specific approach. The five analytes included in the algorithm (and their respective cutoffs) were gp120a (20%), gp160a (35%), gp41a (65%), gp120n (2.8), and gp160n (2.5). The selection was based on the low FRR observed for the 3/5 multi-analyte combination. The cutoff criteria for the 3/5 combination indicates the number of analytes, out of the five candidate analytes, that must exceed the cutoff value established for each respective analyte in order to classify an individual as having progressed from recent to long-term infection.

Given differences in magnitude of avidity and normalized MFI values that do not necessarily reflect biological importance in characterizing recent infection, we evaluated methods for transformation of the 5 highly correlated analytes (Pearson correlations range 0.65–0.88) with a goal to characterize a reduced number of components that represent key construct(s) in the data. Principal component analysis (PCA), a statistical procedure that transforms a set of observations from correlated variables into a set of values of linearly uncorrelated components, was implemented [40, 41]. The resultant component scores are defined as the product of the optimal weight for each original variable and its’ standardized transformation. The PCA score = $\sum_{i=1}^{n}(W_{i}*(\frac{X_{i}-{\hat{X}}_{i}}{S_{i}})$, where n is number of analytes (i = gp160a, gp120a, gp41a, gp160n, gp120n), *W*_*i*_ are analyte PCA weights, *X*_*i*_ are observed analyte values, ${\hat{X}}_{i}$ are analyte means, and *S*_*i*_ are analyte standard deviations. The first principal component explained 81.2% of the variance in the correlated analytes; the variance explained by other components was trivial. Therefore, only the predominant first component was retained for further analyses. The principle component accounted for a greater percentage of analyte variation when all five analytes were included in the formula (data not shown). Similar to PCA, standardization with mean zero and standard deviation of one but with equal weighting per analyte, was implemented so that all five resulting scaled variables could be averaged to create a new score for testing recency. In other words, each analyte contributes equally when calculating the standardized score, as opposed to the PCA score, which assigns a different weight to each analyte. The standardized score = $\sum_{i=1}^{n}(\frac{X_{ij}-{\hat{X}}_{i}}{S_{i}}))/n$, where n is number of analytes (i = gp160a, gp120a, gp41a, gp160n, gp120n), *X*_*ij*_ are observed values for analyte i and person j, ${\hat{X}}_{i}$ are analyte means and S_i_ are analyte standard deviations. In addition to PCA and standardized scores, we also considered the mean value for the three avidity measures (gp120-a, gp160-a, and gp41-a) and the mean value for the two normalized MFI measures (gp120-n and gp160-n) for classifying recent HIV infection.

### Estimation of false-recent rate (FRR)

The FRR is the percent probability that a person infected for longer than 2 years will be misclassified as recently infected by having a measurement value below the selected cutoff value for a given analyte. FRR was calculated from n = 334 specimens collected from persons with known long-term infection. Exact binomial confidence limits (95%) were calculated. To adjust for proportional representation by stage of HIV disease, we also calculated an average FRR across stratum defined by CD4 T-lymphocyte counts. Specimens were classified into four CD4 categories: 0–199 (n = 39), 200–499 (n = 138), 500–799 (n = 93), 800+ (n = 64), with the 38 subjects from our seroconverter panel dataset who were missing CD4 data assigned into the 800+ stratum.

## Results

### Stability of Radix kits

The MFI-n and AI values for the high/ low positive controls over a 12-month period are displayed in Fig 1. The coefficient of variation (CV) of the high positive control for the 12 time points was 15.3% and 13.4% for gp120-n and gp160-n, respectively. For the avidity measures, the CVs for gp120-a, gp160-a, and gp41-a were 1.6%, 3.5%, and 6.4%, respectively. All or the majority of the time points for all five analytes fell within two standard deviations of the mean for the high positive control, with no discernable decline in antibody reactivity over time. For gp41-a, the avidity index value at the nine month time point fell below the 2SD (2 standard deviations) cutoff, however, the avidity index values were within two 2SD of the mean for all remaining time points. The low positive control, included for reference purposes only, remained relatively consistent throughout the evaluation period for all analytes. The mean value for the low positive control was 0.9 and 1.5 for gp120-n and gp160-n, respectively, versus 7.9 and 5.0 for the high positive control. The mean value for the low positive control was 2.6, 5.3, and 2.3% for gp120-a, gp160-a, and gp41-a, respectively, as compared to 61.3, 65.2, and 92.7% for the high positive control.

### MDRI estimates

HIV-1 antibody reactivity for each individual analyte over days since estimated seroconversion is shown in Fig 2. Antibody reactivity, both MFI-n and AI, in general, increased steadily for approximately 200–300 days post-seroconversion, followed by a gradual plateau in antibody responses. The natural variability in biomarker maturation was greater for the gp120 than for gp160 and gp41 antibody responses, as illustrated by the longitudinal analyte values relative to the prediction curve (Fig 2). Of the five algorithms evaluated in this study, multi-analyte, mean normalized MFI (MFI-n), mean avidity index, principle component, and standardized, all but the multi-analyte method generated a single, combined value. For comparison to the individual analytes, the antibody kinetics for the four different analyte algorithms or methods of combining individual analyte values are plotted over days since seroconversion (Fig 3). A similar antibody maturation curve was observed for the combined assay values/scores as compared to the individual analytes. The ability of each individual analyte and algorithm to distinguish recent from long-term infection is demonstrated by the longitudinal curves (Figs 2 and 3). The mean avidity values, principal component and standardized scores had lower residual standard errors, while the mean MFI-n values were comparatively more variable. The principal component and standardized scores can be calculated from the means, standard deviations, and eigenvector of weights given in Table 1.

**Table 1:** Principle component score computation.

	gp120-n	gp160-n	gp120-a	gp160-a	gp41-a
**Mean (**${\bar{X}}_{i}$**)**	2.985	2.667	18.618	31.894	55.546
**Standard Deviation (S**_**i**_**)**	2.045	1.233	15.564	16.635	31.984
**Weight (W**_**i**_**)**	0.448	0.464	0.404	0.451	0.466

The MDRI estimates for each individual analyte and the analyte combinations/algorithms are summarized in Table 2. For the individual analytes, MDRI estimates ranged from 206.5 to 328.5 days, using non-parametric survival methods, and 195.5 to 325.1 days, using the logit random intercept method. The MDRI estimates were similar for both methods, with overlapping 95% confidence limits for all analytes. The MDRI estimates for the five different algorithms, mean MFI, mean AI, multi-analyte algorithm, PCA, and standardized score, ranged from approximately 220 to 270 days and were not markedly different between the two statistical methods.

**Table 2:** Performance characteristics of Multiplex HIV-1 incidence kit.

Biomarker	Cutoff Value	Non-Parametric Survival (95% CL)	Logit Random Intercept (95% CL)	FR/Total LT	Crude FRR (95% CL)	Averaged FRR^b^ (95% CL)
**gp120-n**	2.8	278.2 (228.8, 327.6)	263.6 (218.0, 316.5)	3/334	0.9 (0.2, 2.6)	1.2 (0.2, 7.2)
**gp160-n**	2.5	206.5 (165.6, 247.5)	195.5 (160.2, 235.4)	0/334	0.0 (0.0, 1.1)	0.0 (0.0, 5.3)
**Mean Normalized MFI**	2.6	228.1 (186.7, 269.5)	219.3 (181.5, 261.1)	0/334	0.0 (0.0, 1.1)	0.0 (0.0, 5.3)
**gp120-a**	20	328.5 (278.7, 378.4)	325.1 (277.0, 375.3)	13/334	3.9 (2.1, 6.6)	4.6 (2.0, 11.8)
**gp160-a**	35	272.4 (238.4, 306.3)	262.0 (226.8, 301.2)	1/334	0.3 (0.0, 1.7)	0.2 (0.0, 5.6)
**gp41-a**	65	281.7 (242.3, 321.2)	284.9 (247.7, 326.1)	2/334	0.6 (0.1, 2.1)	0.4 (0.0, 5.9)
**Mean Avidity Index**	40	271.9 (233.7, 310.1)	271.3 (235.3, 312.3)	0/334	0.0 (0.0, 1.1)	0.0 (0.0, 5.3)
**Multi-Analyte Algorithm**^**a**^	3/5 ≥ Respective Cutoff is Non-Recent	250.7 (211.1, 290.3)	243.8 (206.7, 287.8)	0/334	0.0 (0.0, 1.1)	0.0 (0.0, 5.3)
**Principal Component**	0	225.3 (183.7, 266.9)	223.4 (188.6, 264.0)	0/334	0.0 (0.0, 1.1)	0.0 (0.0, 5.3)
**Standardized Score**	0	219.4 (179.1, 259.6)	221.4 (187.3, 260.8)	0/334	0.0 (0.0, 1.1)	0.0 (0.0, 5.3)

^a^Cutoff values: 120a = 20, 160a = 35, 41a = 65, 120n = 2.8, 160n = 2.5.

^b^N = 296 MSM cohort + 38 longitudinal seroconverters; averaged across 4 CD4 strata (assumes Bio-plex observations in 800+ stratum).

### FRR

The FRR for 334 specimens with long-term HIV infection (>2 years) corresponding to each analyte or algorithm is shown in Table 2. The FRR for the individual analytes ranged from 0% to 3.9%, with gp120-a having the highest FRR and the remaining analytes were <1%. To mitigate potential bias in FRR calculations due to overrepresentation of longer-term individuals in the estimates, FRR was also averaged across four CD4+ T cell strata (Table 2). The FRRs were not markedly different between the two estimation methods. For all analyte algorithms, the FRR was 0%, regardless of the specific method used to calculate the estimates.

## Discussion

The MDRI and FRR are critical parameters for all HIV incidence assays, as they impact the estimation of population-based HIV-1 incidence. Previously, we demonstrated the performance characteristics of an in-house developed HIV-1 multiplex assay, based on the Bio-Plex platform, which allows for the use of multi-analyte algorithms for improved estimation of HIV incidence [27]. To facilitate transfer of the assay to other laboratories or testing facilities, the in-house assay was adapted to a kit and a preliminary validation study indicated comparable performance between the in-house assay and kit [28]. Given potential differences in the preparation/manufacturing process of the assay components between the CDC and Radix, characterization of the final kit parameters was warranted. Additionally, statistical methods have evolved since the initial report describing the MDRI estimates for the HIV-1 Multiplex assay [8]. Here, five different algorithms or methods for combining the analytes values were implemented, all of which yielded a 0% FRR with specimens from subtype B infections. Furthermore, we demonstrated stability of the kit components over a one year period, as measured by the consistency in assay values for controls from run-to-run, which is important to demonstrate the transferability of the kit. The assay parameters described in this study provide potential test users with the necessary tools to implement the HIV-1 multiplex assay.

The statistical methods for estimating MDRI have garnered much debate and significant efforts have been made to improve upon these estimates. Recent studies have addressed potential differences in approaches to estimate MDRI by including multiple statistical methods for comparison [8, 30]. Although two different statistical methods were used to estimate the MDRI for each individual analyte and the five algorithms in this study, the MDRIs were not vastly different between the two methods. Similarly, a study evaluating seven different statistical methods for the Limiting Antigen Avidity EIA demonstrated comparable MDRI estimates, which may reflect the careful selection of samples for performing these calculations. To optimize the accuracy of estimates, the longitudinal seroconversion panels included in this study for the estimation of MDRI were selected to minimize the interval of time between last negative and first positive antibody test, minimize the time between sample collections, and maximize total follow-up time.

The MDRI estimates for the individual analytes and all five algorithms were >200 days post-seroconversion, some approaching 300 days. According to the TPP composed by the Incidence Assay Critical Path Working Group, the acceptable MDRI for HIV incidence assay is between 4 to 12 months [2]. Within the acceptable limits of the TPP, the corresponding FRRs were all under 2%, with the exception of gp120-a, and 0% for the intra-assay scores/algorithms. A similar reduction in FRR was observed when various intra-assay algorithms were implemented with the in-house HIV-1 Multiplex assay [27]. Concurrent with previous reports, the antibody response to gp120 was more variable, which is reflected by the higher FRR and longer MDRI [13, 27, 28]. The diversity in the antibody response to gp120 is expected given the sequence and structural variability within the HIV envelope [42]. Despite the variability in the antibody response to gp120, we have demonstrated that the gp120 analyte adds value when incorporated into an algorithm with multiple analytes [27].

Estimates of assay metrics for the algorithms and scores described in this study will require validation for accurate estimation of HIV incidence. However, the use of multiple analytes that are relatively predictive of early HIV infection, transformed into one principal component that accounts for more than 80% of the variability inherent to these key analytes, is a novel approach for estimation of HIV incidence. The inherent variability observed for certain individual analytes is lower for the principal component and standardized scores, which is visually demonstrated in Fig 3. The longitudinal algorithm values follow the prediction curves with limited variation, demonstrating that the algorithms are fairly predictive of time since seroconversion. Misclassification of false recent cases is reduced to near zero. With use of the score values calculated from the descriptive statistics given in Table 2, along with our estimates of MDRI, the multiplex assay can readily be used to estimate population level HIV incidence.

One of the limitations of this study is the number of recent seroconverters with 1–2 years of follow-up at regular intervals available for estimation of the MDRI. Accurate and precise estimates of MDRI are paramount to valid estimation of HIV incidence. Furthermore, given that the FRR may increase with greater cohort/subtype diversity and the study population consisted predominately of subtype B infections, the FRRs described here may not be representative of all populations. Potential differences in the MDRI between subtypes will be addressed in a follow-up study. Although, all five algorithms demonstrated acceptable MDRIs and FRRs, according to the TPP for HIV incidence assays, it is difficult to ascertain which algorithm will be the most useful for field studies and whether there is an advantage of one algorithm over another. All five algorithms exhibited 0% FRR, however, it is not recommended to use an algorithm based solely on titer-based or MFI-n values. Studies have shown that antibody titer is more susceptible to variation in the presence of ART or natural virus suppression [31]. It is likely that the optimal algorithm will include a combination of both antibody titer and avidity [43], which is further illustrated by comparing the variability of the longitudinal mean MFI values (relative to the curve fit) to the algorithms incorporating avidity measures (Fig 3).

In summary, the HIV-1 Multiplex assay may offer added levels of accuracy and precision due to the analytical sensitivity and specificity of the multiplex assay format, the high reproducibility of the kit, and ease in ability to implement intra-assay algorithms in defining of key parameters used in estimation of HIV incidence. The CDC-developed, in-house assay has been produced in kit form to ensure quality control and to facilitate transfer to other laboratories or testing sites. The assay parameters described here, along with availability of a kit, makes the HIV-1 Multiplex assay accessible to external laboratories or testing sites.
